# Corrigendum

**DOI:** 10.1111/1759-7714.14639

**Published:** 2022-09-14

In Zhuo Li *et a*l.^1^ the following error was published on page 3374.

The authors mistakenly used the picture of K510 as NE1 (Figure 2b). Some representative images were misplaced into the manuscript during the stage of figure preparation. Specifically, the mRNA expression level of SULT2B1 in NE1, EC109, EC9706, and KYSE510 cell lines in Figure 2B were incorrect. Our replacement data came from the original data (Original Figure).

**Original Figure.**


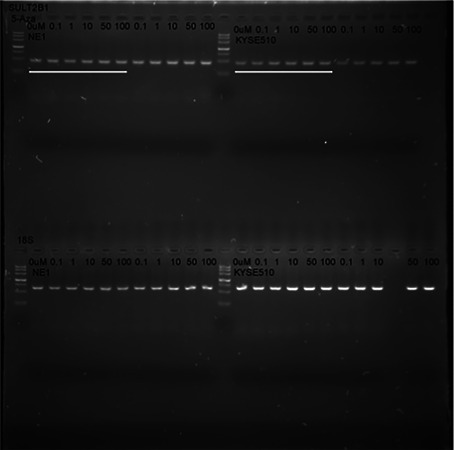


The mRNA expression level of SULT2B1 in NE1 and KYSE510 cells treated with different concentrations of 5‐Aza‐dC (Aza).

The corrected figure panel is reproduced as follow, and the correction does not alter the interpretation of the results and conclusion.


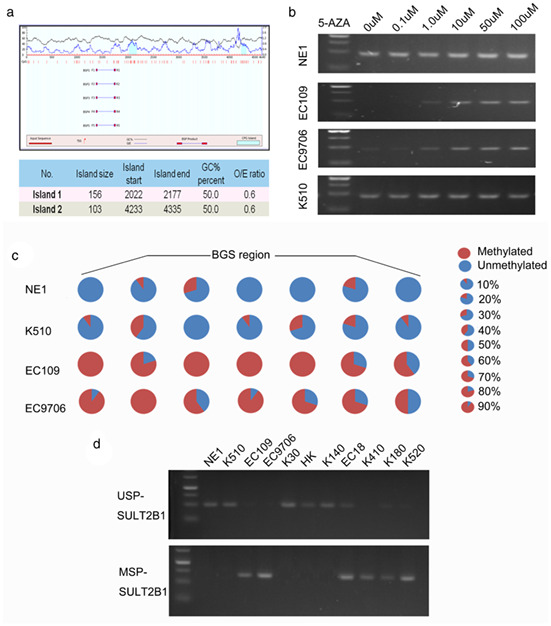


The authors apologize for the error and any inconvenience it may have caused.
